# Supplementary material for: Functional antibodies exhibit light chain coherence
Source: Nature. 2022 Oct 26;611(7935):352–7. doi: 10.1038/s41586-022-05371-z (PMC9607724; doi:10.1038/s41586-022-05371-z)
Supplement: Supplementary file 1 — Supplementary Table 1 describes all data analysed and distributed as part of this work, and how to retrieve them. Supplementary Table 2 exhibits light chain coherence, computed in different ways for several datasets. Supplementary Table 3 displays summary statistics related to junctions within each dataset and recurrence rates within groups of datasets. Supplementary Fig. 1 exhibits associations between Vh and Vl gene usage from 1.2 million single B cells. Supplementary Fig. 2 exhibits per donor CDRH3 length distributions. [file 41586_2022_5371_MOESM1_ESM.pdf]

nature portfolio

https://doi.org/10.1038/s41586-022-05371-z

# Supplementary information

# Functional antibodies exhibit light chain coherence

In the format provided by the authors and unedited

**Supplementary Data for:**

# Functional antibodies exhibit light chain coherence

David B. Jaffe[1,*], Payam Shahi[1,2], Bruce A. Adams[1,3], Ashley M. Chrisman[1,3], Peter M. Finnegan[1,3], Nandhini Raman[1,3], Ariel E. Royall[1,3], FuNien Tsai[1,3], Thomas Vollbrecht[1,3], Daniel S. Reyes[1,2], N. Lance Hepler[2], and Wyatt J. McDonnell[1,*]

[1] 10x Genomics, Inc. 6230 Stoneridge Mall Rd, Pleasanton, CA, 94588. USA.

[2] These authors contributed equally.

[3] These authors contributed equally.

[*] Corresponding authors [99.david.b.jaffe@gmail.com; wyattmcdonnell@gmail.com]

**Supplementary Table 1. All data analyzed and distributed as part of this work.**

| Sample type | Disease | Data identifier(s) to access in enclone | Reference | # of donors | # of cells after filtering |
|---|---|---|---|---|---|
| PBMCs | Healthy donors, longitudinally sampled | phad/phad.meta | Phad et al. | 2 | 247,516 |
| CSF, PBMCs | Multiple sclerosis | part of enclone_paper/scr/other/all.meta | Ramesh et al. | 2 | 8,384 |
| PBMCs | COVID-19 | part of enclone_paper/scr/other/all.meta | Sokal et al. | 5 | 6,361 |
| PBMCs | Healthy, neuromyelitis optica, neurosarcoidosis, RRMS, uveitis | part of enclone_paper/scr/other/all.meta | Ramesh et al. | 1* | 161,836 |
| PBMCs | Kawasaki disease | part of enclone_paper/scr/other/all.meta | Wang et al. | 2* | 17,123 |
| PBMCs | Chronic HIV infection | part of enclone_paper/scr/other/all.meta | Setliff et al. | 2 | 5,391 |
| PBMCs | Healthy donor | 165808, 1017974-1017977, 1018095-1018098, 1018288, 1018296-1018298, 1018301, 1021358, 1021360-1021362, 1029474-1029477, 1031844-1031848, 1031850, 1031851, 1031853, 1031854, 1031856-1031858 | 10x Genomics | 1 | 39,020 |
| Tumor | Ovarian cancer | 123805, 123089, 123547 | 10x Genomics | 1 | 2,888 |
| PBMCs | MALT lymphoma | 83808, 86233 | 10x Genomics | 1 | 9,495 |
| PBMCs | Lupus | 86237 | 10x Genomics | 1 | 252 |
| PBMCs | Healthy donor | 45977 | 10x Genomics | 1 | 8,275 |
| Tumor | Lung cancer | 165807 | 10x Genomics | 1 | 1,191 |
| PBMCs | Healthy donor | 40935 | 10x Genomics | 1 | 17,628 |
| PBMCs | Healthy donor | 52177 | 10x Genomics | 1 | 1,833 |
| PBMCs | Healthy donor | 47201 | 10x Genomics | 1 | 480 |
| PBMCs | Healthy donor | 47202 | 10x Genomics | 1 | 472 |
| PBMCs | Healthy donor | 42787, 42788, 42789 | 10x Genomics | 1 | 40 |
| | **total** | | | **25** | **528,185** |
| PBMCs | Jaffe et al.; 4 donors, this work | @test | This work | | 1,408,939 |
| | **grand total** | | | **29** | **1,937,124** |

* multiple original donors combined

**Supplementary Table 2. Light chain coherence across multiple datasets.** There are three dataset collections which are referred to. We show light chain coherence across these, with varying levels of detail.

**Summary, and commands used to generate items:**

<u>**(a)** data of this work, using all cells in each clonotype</u>
- Command: `public_light_chain_analysis per_cell_stuff`

| Memory B cells, all cells and clonotypes | | | | | | | | |
|---|---|---|---|---|---|---|---|---|
| CDRH3 amino acid % identity | $log_{10}$ of cell pairs | Donors and percent light chain coherence | | | | | | |
| | | any | 1,2 | 1,3 | 1,4 | 2,3 | 2,4 | 3,4 |
| 0% | 5.6 | 5 | 4 | 5 | 5 | 4 | 4 | 5 |
| 10% | 7.4 | 5 | 5 | 5 | 5 | 5 | 5 | 5 |
| 20% | 8.2 | 5 | 5 | 5 | 5 | 5 | 5 | 5 |
| 30% | 8.3 | 5 | 5 | 6 | 5 | 5 | 5 | 5 |
| 40% | 8.0 | 6 | 6 | 6 | 6 | 5 | 5 | 6 |
| 50% | 7.6 | 7 | 7 | 8 | 7 | 7 | 6 | 6 |
| 60% | 6.7 | 13 | 14 | 14 | 13 | 11 | 10 | 11 |
| 70% | 5.8 | 31 | 38 | 30 | 30 | 27 | 25 | 25 |
| 80% | 5.2 | 62 | 62 | 62 | 67 | 54 | 56 | 57 |
| 90% | 4.3 | 75 | 66 | 78 | 79 | 79 | 72 | 75 |
| 100% | 3.7 | 82 | 71 | 69 | 92 | 91 | 85 | 72 |

| Naive B cells, all cells and clonotypes | | | | | | | | |
|---|---|---|---|---|---|---|---|---|
| CDRH3 amino acid % identity | $log_{10}$ of cell pairs | Donors and percent light chain coherence | | | | | | |
| | | any | 1,2 | 1,3 | 1,4 | 2,3 | 2,4 | 3,4 |
| 0% | 4.5 | 4 | 4 | 4 | 4 | 4 | 3 | 4 |
| 10% | 7.0 | 5 | 5 | 5 | 5 | 4 | 5 | 4 |
| 20% | 8.2 | 5 | 5 | 5 | 5 | 5 | 5 | 5 |
| 30% | 8.4 | 5 | 5 | 5 | 5 | 5 | 5 | 5 |
| 40% | 8.2 | 5 | 5 | 5 | 5 | 5 | 5 | 5 |
| 50% | 7.8 | 5 | 5 | 6 | 6 | 5 | 5 | 5 |
| 60% | 7.1 | 6 | 6 | 6 | 6 | 6 | 6 | 6 |
| 70% | 6.3 | 6 | 6 | 7 | 6 | 6 | 6 | 6 |
| 80% | 5.3 | 7 | 7 | 7 | 7 | 7 | 6 | 7 |
| 90% | 3.9 | 8 | 7 | 9 | 8 | 7 | 8 | 8 |
| 100% | 2.6 | 10 | 9 | 7 | 12 | 9 | 10 | 10 |

| Cell pair counts, $log_{10}$, memory B cells; all cells | | | | | | | |
|---|---|---|---|---|---|---|---|
| CDRH3-AA % identity | any | d1, d2 | d1, d3 | d1, d4 | d2, d3 | d2, d4 | d3, d4 |
| 0% | 5.6 | 4.9 | 5.0 | 5.1 | 4.5 | 4.5 | 4.6 |
| 10% | 7.4 | 6.6 | 6.7 | 6.8 | 6.3 | 6.4 | 6.5 |
| 20% | 8.2 | 7.4 | 7.5 | 7.6 | 7.1 | 7.2 | 7.3 |
| 30% | 8.3 | 7.6 | 7.6 | 7.8 | 7.2 | 7.3 | 7.4 |
| 40% | 8.0 | 7.3 | 7.4 | 7.5 | 6.9 | 7.1 | 7.1 |
| 50% | 7.6 | 6.8 | 6.9 | 7.0 | 6.4 | 6.6 | 6.6 |
| 60% | 6.7 | 6.0 | 6.1 | 6.2 | 5.6 | 5.8 | 5.8 |
| 70% | 5.8 | 5.2 | 5.2 | 5.3 | 4.7 | 4.8 | 4.8 |
| 80% | 5.2 | 4.5 | 4.5 | 4.7 | 3.9 | 4.1 | 4.1 |
| 90% | 4.3 | 3.7 | 3.6 | 3.9 | 3.1 | 3.4 | 3.2 |
| 100% | 3.7 | 3.2 | 2.7 | 3.4 | 2.3 | 2.7 | 2.5 |

| Cell pair counts, $log_{10}$, naive B cells; all cells | | | | | | | |
|---|---|---|---|---|---|---|---|
| CDRH3-AA % identity | any | d1, d2 | d1, d3 | d1, d4 | d2, d3 | d2, d4 | d3, d4 |
| 0% | 4.5 | 3.5 | 3.6 | 3.7 | 3.8 | 3.9 | 3.9 |
| 10% | 7.0 | 6.0 | 6.1 | 6.1 | 6.4 | 6.3 | 6.4 |
| 20% | 8.2 | 7.2 | 7.2 | 7.3 | 7.5 | 7.5 | 7.5 |
| 30% | 8.4 | 7.4 | 7.4 | 7.5 | 7.7 | 7.8 | 7.8 |
| 40% | 8.2 | 7.2 | 7.2 | 7.3 | 7.5 | 7.6 | 7.5 |
| 50% | 7.8 | 6.9 | 6.8 | 7.0 | 7.1 | 7.2 | 7.2 |
| 60% | 7.1 | 6.3 | 6.1 | 6.3 | 6.3 | 6.6 | 6.4 |
| 70% | 6.3 | 5.4 | 5.2 | 5.5 | 5.4 | 5.7 | 5.5 |
| 80% | 5.3 | 4.4 | 4.3 | 4.5 | 4.4 | 4.8 | 4.6 |
| 90% | 3.9 | 3.0 | 2.9 | 3.1 | 3.0 | 3.3 | 3.2 |
| 100% | 2.6 | 1.8 | 1.6 | 1.8 | 1.7 | 2.1 | 1.9 |

| Cell counts, $log_{10}$, memory B cells; all cells | | | | | | | |
|---|---|---|---|---|---|---|---|
| CDRH3-AA % identity | any | d1, d2 | d1, d3 | d1, d4 | d2, d3 | d2, d4 | d3, d4 |
| 0% | 5.1 | 4.5 | 4.6 | 4.6 | 4.2 | 4.2 | 4.4 |
| 10% | 5.8 | 5.4 | 5.5 | 5.5 | 5.2 | 5.3 | 5.3 |
| 20% | 5.8 | 5.5 | 5.6 | 5.6 | 5.3 | 5.4 | 5.4 |
| 30% | 5.8 | 5.5 | 5.6 | 5.6 | 5.3 | 5.4 | 5.4 |
| 40% | 5.8 | 5.5 | 5.5 | 5.6 | 5.3 | 5.3 | 5.4 |
| 50% | 5.7 | 5.5 | 5.5 | 5.5 | 5.2 | 5.3 | 5.3 |
| 60% | 5.6 | 5.2 | 5.3 | 5.3 | 5.0 | 5.1 | 5.1 |
| 70% | 5.2 | 4.8 | 4.8 | 4.9 | 4.4 | 4.6 | 4.6 |
| 80% | 4.7 | 4.2 | 4.2 | 4.3 | 3.8 | 4.0 | 3.9 |
| 90% | 4.0 | 3.5 | 3.4 | 3.5 | 3.1 | 3.2 | 3.1 |
| 100% | 3.4 | 3.0 | 2.7 | 3.0 | 2.3 | 2.6 | 2.4 |

| Cell counts, $log_{10}$, naive B cells; all cells | | | | | | | |
|---|---|---|---|---|---|---|---|
| CDRH3-AA % identity | any | d1, d2 | d1, d3 | d1, d4 | d2, d3 | d2, d4 | d3, d4 |
| 0% | 4.4 | 3.5 | 3.5 | 3.6 | 3.8 | 3.8 | 3.9 |
| 10% | 5.8 | 5.3 | 5.3 | 5.3 | 5.5 | 5.5 | 5.5 |
| 20% | 5.9 | 5.5 | 5.5 | 5.5 | 5.6 | 5.6 | 5.6 |
| 30% | 5.9 | 5.5 | 5.5 | 5.5 | 5.6 | 5.6 | 5.7 |
| 40% | 5.9 | 5.5 | 5.5 | 5.5 | 5.6 | 5.6 | 5.6 |
| 50% | 5.9 | 5.5 | 5.5 | 5.5 | 5.6 | 5.6 | 5.6 |
| 60% | 5.8 | 5.4 | 5.4 | 5.4 | 5.5 | 5.5 | 5.5 |
| 70% | 5.6 | 5.1 | 5.0 | 5.1 | 5.1 | 5.3 | 5.2 |
| 80% | 5.2 | 4.5 | 4.4 | 4.5 | 4.5 | 4.7 | 4.6 |
| 90% | 4.1 | 3.2 | 3.2 | 3.4 | 3.3 | 3.6 | 3.4 |
| 100% | 2.9 | 2.0 | 1.9 | 2.1 | 2.0 | 2.3 | 2.2 |

**(b)** data of this work, using one cell per clonotype

- Command: `public_light_chain_analysis per_cell_stuff SOLO`

Note that for naive cells, results are almost identical to **(a)** because clonotypes for naive cells almost always contain just one cell.

| Memory B cells; 1 cell per clonotype | | | | | | | | |
|---|---|---|---|---|---|---|---|---|
| CDRH3 amino acid % identity | $log_{10}$ of cell pairs | Donors and percent light chain coherence | | | | | | |
| | | any | 1,2 | 1,3 | 1,4 | 2,3 | 2,4 | 3,4 |
| 0% | 5.2 | 5 | 5 | 5 | 5 | 5 | 4 | 5 |
| 10% | 7.0 | 5 | 5 | 5 | 5 | 4 | 5 | 5 |
| 20% | 7.8 | 5 | 5 | 5 | 5 | 5 | 5 | 5 |
| 30% | 8.0 | 5 | 5 | 5 | 5 | 5 | 5 | 5 |
| 40% | 7.7 | 5 | 6 | 6 | 5 | 5 | 5 | 5 |
| 50% | 7.3 | 6 | 6 | 7 | 6 | 6 | 6 | 6 |
| 60% | 6.4 | 8 | 9 | 10 | 8 | 9 | 7 | 8 |
| 70% | 5.5 | 17 | 19 | 18 | 16 | 18 | 15 | 15 |
| 80% | 4.6 | 38 | 42 | 37 | 41 | 38 | 35 | 33 |
| 90% | 3.7 | 63 | 62 | 61 | 65 | 63 | 64 | 58 |
| 100% | 3.0 | 79 | 74 | 74 | 84 | 80 | 83 | 75 |

| Naive B cells; 1 cell per clonotype | | | | | | | | |
|---|---|---|---|---|---|---|---|---|
| CDRH3 amino acid % identity | $log_{10}$ of cell pairs | Donors and percent light chain coherence | | | | | | |
| | | any | 1,2 | 1,3 | 1,4 | 2,3 | 2,4 | 3,4 |
| 0% | 4.5 | 4 | 4 | 4 | 4 | 4 | 4 | 4 |
| 10% | 7.0 | 5 | 5 | 5 | 5 | 4 | 5 | 4 |
| 20% | 8.2 | 5 | 5 | 5 | 5 | 5 | 5 | 5 |
| 30% | 8.4 | 5 | 5 | 5 | 5 | 5 | 5 | 5 |
| 40% | 8.2 | 5 | 5 | 5 | 5 | 5 | 5 | 5 |
| 50% | 7.8 | 5 | 5 | 6 | 6 | 5 | 5 | 5 |
| 60% | 7.1 | 6 | 6 | 6 | 6 | 6 | 6 | 6 |
| 70% | 6.3 | 6 | 6 | 6 | 6 | 6 | 6 | 6 |
| 80% | 5.3 | 7 | 7 | 7 | 7 | 7 | 6 | 7 |
| 90% | 3.9 | 8 | 7 | 9 | 9 | 7 | 8 | 8 |
| 100% | 2.6 | 10 | 9 | 7 | 10 | 9 | 10 | 10 |

| Cell pair counts, $log_{10}$, memory B cells; 1 cell | | | | | | | |
|---|---|---|---|---|---|---|---|
| CDRH3-AA % identity | any | d1, d2 | d1, d3 | d1, d4 | d2, d3 | d2, d4 | d3, d4 |
| 0% | 5.2 | 4.4 | 4.6 | 4.6 | 4.1 | 4.1 | 4.3 |
| 10% | 7.0 | 6.2 | 6.3 | 6.4 | 6.0 | 6.0 | 6.1 |
| 20% | 7.8 | 7.1 | 7.2 | 7.2 | 6.9 | 6.9 | 7.0 |
| 30% | 8.0 | 7.2 | 7.3 | 7.4 | 7.0 | 7.1 | 7.1 |
| 40% | 7.7 | 7.0 | 7.0 | 7.1 | 6.7 | 6.8 | 6.8 |
| 50% | 7.3 | 6.5 | 6.6 | 6.7 | 6.2 | 6.4 | 6.4 |
| 60% | 6.4 | 5.7 | 5.8 | 5.8 | 5.4 | 5.5 | 5.5 |
| 70% | 5.5 | 4.7 | 4.8 | 4.9 | 4.4 | 4.6 | 4.6 |
| 80% | 4.6 | 3.9 | 4.0 | 4.0 | 3.6 | 3.7 | 3.7 |
| 90% | 3.7 | 3.1 | 3.0 | 3.1 | 2.6 | 2.9 | 2.7 |
| 100% | 3.0 | 2.5 | 2.1 | 2.5 | 1.8 | 2.2 | 2.0 |

| Cell pair counts, $log_{10}$, naive B cells; 1 cell | | | | | | | |
|---|---|---|---|---|---|---|---|
| CDRH3-AA % identity | any | d1, d2 | d1, d3 | d1, d4 | d2, d3 | d2, d4 | d3, d4 |
| 0% | 4.5 | 3.4 | 3.6 | 3.6 | 3.8 | 3.8 | 3.9 |
| 10% | 7.0 | 6.0 | 6.1 | 6.1 | 6.4 | 6.3 | 6.4 |
| 20% | 8.2 | 7.2 | 7.2 | 7.2 | 7.5 | 7.5 | 7.5 |
| 30% | 8.4 | 7.4 | 7.4 | 7.5 | 7.7 | 7.7 | 7.8 |
| 40% | 8.2 | 7.2 | 7.2 | 7.3 | 7.5 | 7.6 | 7.5 |
| 50% | 7.8 | 6.9 | 6.8 | 7.0 | 7.1 | 7.2 | 7.2 |
| 60% | 7.1 | 6.2 | 6.1 | 6.3 | 6.3 | 6.6 | 6.4 |
| 70% | 6.3 | 5.4 | 5.2 | 5.4 | 5.4 | 5.7 | 5.5 |
| 80% | 5.3 | 4.4 | 4.3 | 4.5 | 4.4 | 4.8 | 4.6 |
| 90% | 3.9 | 3.0 | 2.9 | 3.1 | 3.0 | 3.3 | 3.2 |
| 100% | 2.6 | 1.8 | 1.6 | 1.8 | 1.7 | 2.1 | 1.9 |

| Cell counts, $log_{10}$, memory B cells; 1 cell | | | | | | | |
|---|---|---|---|---|---|---|---|
| CDRH3-AA % identity | any | d1, d2 | d1, d3 | d1, d4 | d2, d3 | d2, d4 | d3, d4 |
| 0% | 4.9 | 4.2 | 4.4 | 4.4 | 4.0 | 4.0 | 4.2 |
| 10% | 5.6 | 5.2 | 5.3 | 5.3 | 5.1 | 5.1 | 5.2 |
| 20% | 5.6 | 5.3 | 5.4 | 5.4 | 5.2 | 5.2 | 5.3 |
| 30% | 5.6 | 5.3 | 5.4 | 5.4 | 5.2 | 5.2 | 5.3 |
| 40% | 5.6 | 5.3 | 5.4 | 5.4 | 5.2 | 5.2 | 5.3 |
| 50% | 5.6 | 5.3 | 5.3 | 5.3 | 5.1 | 5.2 | 5.2 |
| 60% | 5.4 | 5.1 | 5.1 | 5.1 | 4.9 | 4.9 | 4.9 |
| 70% | 5.1 | 4.6 | 4.6 | 4.7 | 4.3 | 4.5 | 4.4 |
| 80% | 4.5 | 3.9 | 4.0 | 4.0 | 3.6 | 3.8 | 3.7 |
| 90% | 3.7 | 3.2 | 3.1 | 3.2 | 2.8 | 3.0 | 2.9 |
| 100% | 3.1 | 2.6 | 2.3 | 2.6 | 2.0 | 2.3 | 2.2 |

| Cell counts, $log_{10}$, naive B cells; 1 cell | | | | | | | |
|---|---|---|---|---|---|---|---|
| CDRH3-AA % identity | any | d1, d2 | d1, d3 | d1, d4 | d2, d3 | d2, d4 | d3, d4 |
| 0% | 4.4 | 3.4 | 3.5 | 3.6 | 3.7 | 3.8 | 3.8 |
| 10% | 5.8 | 5.3 | 5.3 | 5.3 | 5.5 | 5.5 | 5.5 |
| 20% | 5.9 | 5.5 | 5.5 | 5.5 | 5.6 | 5.6 | 5.6 |
| 30% | 5.9 | 5.5 | 5.5 | 5.5 | 5.6 | 5.6 | 5.6 |
| 40% | 5.9 | 5.5 | 5.5 | 5.5 | 5.6 | 5.6 | 5.6 |
| 50% | 5.9 | 5.5 | 5.5 | 5.5 | 5.6 | 5.6 | 5.6 |
| 60% | 5.8 | 5.4 | 5.4 | 5.4 | 5.5 | 5.5 | 5.5 |
| 70% | 5.6 | 5.0 | 5.0 | 5.1 | 5.1 | 5.3 | 5.2 |
| 80% | 5.2 | 4.5 | 4.4 | 4.5 | 4.5 | 4.7 | 4.6 |
| 90% | 4.1 | 3.2 | 3.2 | 3.3 | 3.3 | 3.6 | 3.4 |
| 100% | 2.9 | 2.0 | 1.9 | 2.1 | 2.0 | 2.3 | 2.2 |

**(c)** older data, using all cells in each clonotype

- Command: `public_light_chain_analysis per_cell_stuff.old_data MANY`

| Memory B cells; all cells and clonotypes | | |
|---|---|---|
| CDRH3 amino acid % identity | $\log_{10}$ of cell pairs | Donors and percent light chain coherence |
| | | any |
| 0% | 4.1 | 4 |
| 10% | 6.0 | 4 |
| 20% | 6.9 | 5 |
| 30% | 7.0 | 5 |
| 40% | 6.7 | 6 |
| 50% | 6.2 | 7 |
| 60% | 5.4 | 9 |
| 70% | 4.4 | 19 |
| 80% | 3.6 | 38 |
| 90% | 2.8 | 67 |
| 100% | 2.4 | 93 |

| Naive B cells; all cells and clonotypes | | |
|---|---|---|
| CDRH3 amino acid % identity | $\log_{10}$ of cell pairs | Donors and percent light chain coherence |
| | | any |
| 0% | 2.5 | 4 |
| 10% | 5.3 | 4 |
| 20% | 6.5 | 4 |
| 30% | 6.7 | 5 |
| 40% | 6.5 | 5 |
| 50% | 6.1 | 5 |
| 60% | 5.5 | 6 |
| 70% | 4.6 | 6 |
| 80% | 3.7 | 8 |
| 90% | 2.3 | 5 |
| 100% | 1.4 | 17 |

**(d)** older data, using one cell per clonotype

- Command: `public_light_chain_analysis per_cell_stuff.old_data MANY SOLO`

| Memory B cells; 1 cell per clonotype | | |
|---|---|---|
| CDRH3 amino acid % identity | $\log_{10}$ of cell pairs | Donors and percent light chain coherence |
| | | any |
| 0% | 4.0 | 5 |
| 10% | 5.9 | 4 |
| 20% | 6.7 | 4 |
| 30% | 6.8 | 5 |
| 40% | 6.6 | 5 |
| 50% | 6.1 | 5 |
| 60% | 5.2 | 7 |
| 70% | 4.3 | 16 |
| 80% | 3.4 | 40 |
| 90% | 2.6 | 60 |
| 100% | 2.1 | 87 |

| Naive B cells; 1 cell per clonotype | | |
|---|---|---|
| CDRH3 amino acid % identity | $\log_{10}$ of cell pairs | Donors and percent light chain coherence |
| | | any |
| 0% | 2.5 | 4 |
| 10% | 5.3 | 4 |
| 20% | 6.5 | 4 |
| 30% | 6.7 | 5 |
| 40% | 6.5 | 5 |
| 50% | 6.1 | 5 |
| 60% | 5.5 | 5 |
| 70% | 4.6 | 6 |
| 80% | 3.7 | 7 |
| 90% | 2.2 | 5 |
| 100% | 1.3 | 18 |

**(e)** Phad 2022 data, using all cells in each clonotype

- Command: `public_light_chain_analysis light_chain_coherence/results/per_cell_stuff.phad MANY`

| Memory B cells; all cells and clonotypes | | |
|---|---|---|
| CDRH3 amino acid % identity | $\log_{10}$ of cell pairs | Donors and percent light chain coherence: any |
| 0% | 4.8 | 4 |
| 10% | 6.5 | 5 |
| 20% | 7.3 | 5 |
| 30% | 7.4 | 5 |
| 40% | 7.1 | 6 |
| 50% | 6.6 | 7 |
| 60% | 5.6 | 12 |
| 70% | 4.7 | 36 |
| 80% | 4.1 | 63 |
| 90% | 3.5 | 87 |
| 100% | 3.0 | 79 |

| Naive B cells; all cells and clonotypes | | |
|---|---|---|
| CDRH3 amino acid % identity | $\log_{10}$ of cell pairs | Donors and percent light chain coherence: any |
| 0% | 0.0 | 0 |
| 10% | 2.6 | 4 |
| 20% | 3.8 | 5 |
| 30% | 4.0 | 5 |
| 40% | 3.8 | 5 |
| 50% | 3.5 | 5 |
| 60% | 2.7 | 5 |
| 70% | 1.8 | 1 |
| 80% | 0.8 | 0 |
| 90% | -- | -- |
| 100% | -- | -- |

**(f)** Phad 2022 data, using one cell per clonotype

- Command: `public_light_chain_analysis light_chain_coherence/results/per_cell_stuff.phad MANY SOLO`

| Memory B cells; 1 cell per clonotype | | |
|---|---|---|
| CDRH3 amino acid % identity | $\log_{10}$ of cell pairs | Donors and percent light chain coherence: any |
| 0% | 4.0 | 4 |
| 10% | 5.9 | 5 |
| 20% | 6.7 | 5 |
| 30% | 6.8 | 5 |
| 40% | 6.5 | 5 |
| 50% | 6.0 | 6 |
| 60% | 5.1 | 8 |
| 70% | 4.0 | 19 |
| 80% | 3.2 | 45 |
| 90% | 2.3 | 64 |
| 100% | 1.8 | 69 |

| Naive B cells; 1 cell per clonotype | | |
|---|---|---|
| CDRH3 amino acid % identity | $\log_{10}$ of cell pairs | Donors and percent light chain coherence: any |
| 0% | 0.0 | 0 |
| 10% | 2.5 | 4 |
| 20% | 3.7 | 5 |
| 30% | 3.9 | 5 |
| 40% | 3.7 | 5 |
| 50% | 3.4 | 5 |
| 60% | 2.7 | 6 |
| 70% | 1.8 | 2 |
| 80% | 0.8 | 0 |
| 90% | -- | -- |
| 100% | -- | -- |

**(g)** all data, each dataset collection treated as one donor, all cells in each clonotype

- Command: `public_light_chain_analysis per_cell_stuff,per_cell_stuff.phad,per_cell_stuff.old_data`

| Memory B cells; all cells and clonotypes | | | | | |
|---|---|---|---|---|---|
| CDRH3 amino acid % identity | $\log_{10}$ of cell pairs | Datasets and percent light chain coherence | | | |
| | | any | 1,2 | 1,3 | 2,3 |
| 0% | 6.0 | 4 | 4 | 5 | 4 |
| 10% | 7.8 | 5 | 5 | 5 | 5 |
| 20% | 8.5 | 5 | 5 | 5 | 5 |
| 30% | 8.7 | 5 | 5 | 5 | 5 |
| 40% | 8.4 | 6 | 6 | 6 | 6 |
| 50% | 7.9 | 7 | 8 | 6 | 6 |
| 60% | 7.0 | 12 | 13 | 11 | 10 |
| 70% | 6.1 | 29 | 31 | 27 | 28 |
| 80% | 5.4 | 55 | 56 | 53 | 56 |
| 90% | 4.6 | 69 | 67 | 71 | 71 |
| 100% | 4.0 | 70 | 58 | 86 | 82 |

| Naive B cells; all cells and clonotypes | | | | | |
|---|---|---|---|---|---|
| CDRH3 amino acid % identity | $\log_{10}$ of cell pairs | Datasets and percent light chain coherence | | | |
| | | any | 1,2 | 1,3 | 2,3 |
| 0% | 4.0 | 4 | 5 | 4 | 7 |
| 10% | 6.6 | 4 | 5 | 4 | 5 |
| 20% | 7.8 | 5 | 5 | 5 | 5 |
| 30% | 8.0 | 5 | 5 | 5 | 5 |
| 40% | 7.8 | 5 | 5 | 5 | 5 |
| 50% | 7.4 | 5 | 5 | 5 | 5 |
| 60% | 6.8 | 6 | 6 | 6 | 5 |
| 70% | 5.9 | 6 | 6 | 6 | 7 |
| 80% | 5.0 | 6 | 8 | 6 | 6 |
| 90% | 3.5 | 8 | 8 | 8 | 13 |
| 100% | 2.2 | 15 | 25 | 15 | -- |

**(h)** all data, each dataset collection treated as one donor, one cell per clonotype

`public_light_chain_analysis per_cell_stuff,per_cell_stuff.phad,per_cell_stuff.old_data SOLO`

| Memory B cells; 1 cell per clonotype | | | | | |
|---|---|---|---|---|---|
| CDRH3 amino acid % identity | $\log_{10}$ of cell pairs | Datasets and percent light chain coherence | | | |
| | | any | 1,2 | 1,3 | 2,3 |
| 0% | 5.5 | 5 | 5 | 5 | 5 |
| 10% | 7.4 | 5 | 5 | 5 | 5 |
| 20% | 8.2 | 5 | 5 | 5 | 5 |
| 30% | 8.3 | 5 | 5 | 5 | 5 |
| 40% | 8.0 | 5 | 5 | 5 | 5 |
| 50% | 7.5 | 6 | 6 | 6 | 6 |
| 60% | 6.7 | 8 | 8 | 8 | 8 |
| 70% | 5.7 | 17 | 16 | 17 | 17 |
| 80% | 4.9 | 39 | 37 | 39 | 42 |
| 90% | 4.0 | 62 | 59 | 63 | 63 |
| 100% | 3.3 | 77 | 76 | 80 | 73 |

| Naive B cells; 1 cell per clonotype | | | | | |
|---|---|---|---|---|---|
| CDRH3 amino acid % identity | $\log_{10}$ of cell pairs | Datasets and percent light chain coherence | | | |
| | | any | 1,2 | 1,3 | 2,3 |
| 0% | 4.0 | 4 | 5 | 4 | 8 |
| 10% | 6.6 | 4 | 5 | 4 | 5 |
| 20% | 7.8 | 5 | 5 | 5 | 5 |
| 30% | 8.0 | 5 | 5 | 5 | 5 |
| 40% | 7.8 | 5 | 5 | 5 | 5 |
| 50% | 7.4 | 5 | 5 | 5 | 5 |
| 60% | 6.8 | 6 | 6 | 6 | 5 |
| 70% | 5.9 | 6 | 6 | 6 | 7 |
| 80% | 4.9 | 7 | 7 | 6 | 6 |
| 90% | 3.5 | 8 | 7 | 8 | 9 |
| 100% | 2.2 | 13 | 25 | 13 | -- |

(i) data of this work, using all cells in each clonotype, comparing within donors (**Figure 1b**)

```
private_light_chain_analysis per_cell_stuff
```

| Memory B cells | | | | | | |
|---|---|---|---|---|---|---|
| CDRH3 amino acid % identity | $\log_{10}$ of cell pairs | Donors and percent light chain coherence | | | | |
| | | All | 1 | 2 | 3 | 4 |
| 0% | 4.8 | 4 | 5 | 4 | 5 | 3 |
| 10% | 6.5 | 5 | 5 | 4 | 4 | 4 |
| 20% | 7.4 | 5 | 5 | 4 | 5 | 5 |
| 30% | 7.6 | 5 | 5 | 5 | 5 | 5 |
| 40% | 7.3 | 5 | 5 | 5 | 5 | 5 |
| 50% | 6.9 | 6 | 6 | 5 | 5 | 5 |
| 60% | 6.1 | 8 | 9 | 7 | 7 | 6 |
| 70% | 5.2 | 15 | 18 | 14 | 12 | 12 |
| 80% | 4.3 | 36 | 42 | 33 | 25 | 32 |
| 90% | 3.5 | 60 | 61 | 55 | 55 | 62 |
| 100% | 3.0 | 65 | 66 | 70 | 66 | 62 |

| Naive B cells | | | | | | |
|---|---|---|---|---|---|---|
| CDRH3 amino acid % identity | $\log_{10}$ of cell pairs | Donors and percent light chain coherence | | | | |
| | | All | 1 | 2 | 3 | 4 |
| 0% | 4.1 | 4 | 4 | 3 | 4 | 4 |
| 10% | 6.6 | 5 | 5 | 5 | 5 | 5 |
| 20% | 7.8 | 5 | 5 | 5 | 5 | 5 |
| 30% | 8.0 | 5 | 5 | 5 | 5 | 5 |
| 40% | 7.8 | 5 | 5 | 5 | 5 | 5 |
| 50% | 7.5 | 5 | 5 | 5 | 5 | 5 |
| 60% | 6.8 | 5 | 6 | 5 | 6 | 5 |
| 70% | 6.0 | 6 | 6 | 6 | 6 | 6 |
| 80% | 5.0 | 6 | 8 | 6 | 6 | 6 |
| 90% | 3.6 | 8 | 11 | 7 | 8 | 8 |
| 100% | 2.5 | 12 | 16 | 12 | 12 | 10 |

| Data source | Simulation parameters | | Average junction property | | | | | | |
|---|---|---|---|---|---|---|---|---|---|
| | Unique sequences | Selection model | VDJ recurrences (# ± SEM) | VDDJ recurrences (# ± SEM) | Insertion length (NT) | Substitution rate | Substitution rate (cells w/ 0 insertions) | VDDJ rate | CDRH3 length (AA) |
| Real | -- | -- | 754.0 ( N/A ) | 0.0 (N/A ) | 5.0 | 15.6% | 7.2% | 0.52% | 18.4 |
| Naive | No | Post | 1189.6 (±10.8) | 1.8 (±0.7) | 5.3 | 16.5% | 7.3% | 0.32% | 18.1 |
| Naive | Yes | Post | 1217.3 (±16.9) | 0.6 (±0.3) | 5.4 | 16.6% | 7.3% | 0.32% | 18.1 |
| Memory | No | Post | 1444.5 (±13.8) | 1.0 (±0.3) | 4.8 | 17.4% | 7.7% | 0.35% | 17.4 |
| Memory | Yes | Post | 1632.0 (±14.8) | 1.6 (±0.6) | 4.6 | 17.5% | 7.7% | 0.28% | 17.2 |
| Naive | No | Pre | 167.4 (± 5.8) | 1.4 (±0.6) | 8.7 | 16.7% | 8.3% | 1.32% | 21.0 |
| Naive | Yes | Pre | 172.1 (±. 3.6) | 1.0 (±0.4) | 8.8 | 16.7% | 8.8% | 1.30% | 21.0 |
| Memory | No | Pre | 173.3 (±. 6.1) | 1.0 (±0.3) | 8.8 | 16.8% | 8.3% | 1.34% | 21.0 |
| Memory | Yes | Pre | 175.4 (±. 4.5) | 2.0 (±0.6) | 8.7 | 16.7% | 8.3% | 1.35% | 21.1 |

**Supplementary Table 3. A comparison of repertoire properties and recurrence rates for multiple simulation settings.** We examined the effects of training soNNia models using naive sequences vs. memory sequences for training data, whether the training data were deduplicated (unique) or used all observed sequences from all cells, and using preselection vs. postselection models.

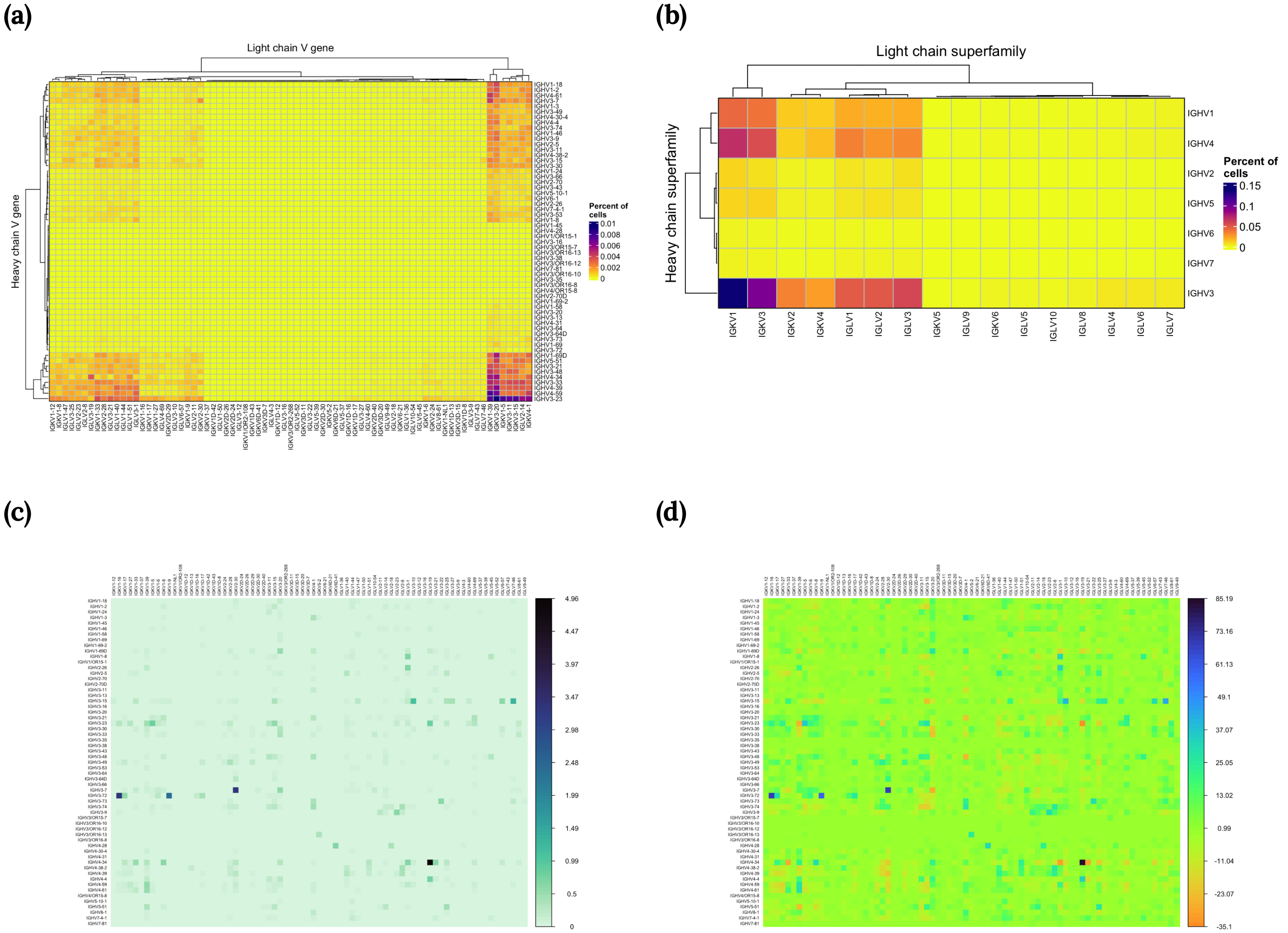


**Supplementary Figure 1. Associations between $V_H$ and $V_L$ gene usage from 1.2 million single B cells.** Using unique CDR3 sequences (n=1,214,376) from all donors, we tabulated $V_H$ and $V_L$ gene usage. We then performed a two-sided chi-squared test and calculated its significance via Monte Carlo simulation ($10^5$ bootstraps, p = 0.00008). This provides evidence of statistically significant associations between $V_H$ and $V_L$ genes. **(a)** Gene-level associations between $V_H$ and $V_L$ genes. **(b)** Superfamily-level associations between $V_H$ and $V_L$ genes. and b at the superfamily and gene levels respectively. **(c)** Heatmap of the residuals of the chi-squared test, visualizing important associations that contributed to the significance of the test. **(d)** Heatmap of the contributions of the residuals of the chi-squared test, visualizing important associations that contributed to the significance of the test.

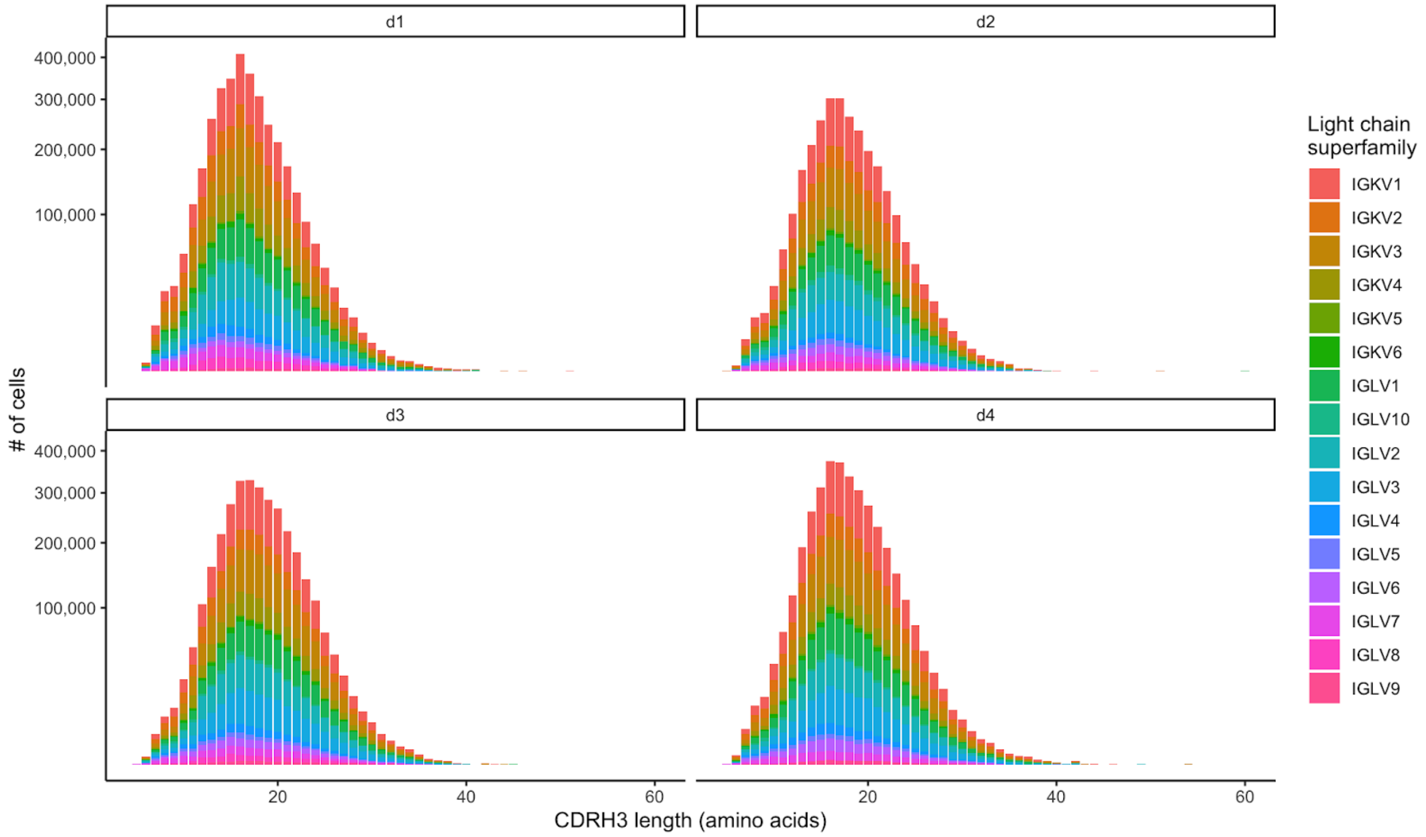


**Supplementary Figure 2. Per-donor CDRH3 length distributions.** Each histogram barplot is fractionally colored by the number of cells with a given light chain V gene superfamily.
