# Supplementary material for: Functional antibodies exhibit light chain coherence
Source: Nature. 2022 Oct 26;611(7935):352–7. doi: 10.1038/s41586-022-05371-z (PMC9607724; doi:10.1038/s41586-022-05371-z)
Supplement: Supplementary file 2 — Reporting Summary [file 41586_2022_5371_MOESM2_ESM.pdf]

nature portfolio

Corresponding author(s): David B. Jaffe, Wyatt J. McDonnell

Last updated by author(s): Sep 10, 2022

Reporting Summary

Nature Portfolio wishes to improve the reproducibility of the work that we publish. This form provides structure for consistency and transparency in reporting. For further information on Nature Portfolio policies, see our Editorial Policies and the Editorial Policy Checklist.

Statistics

For all statistical analyses, confirm that the following items are present in the figure legend, table legend, main text, or Methods section.

n/a	Confirmed	
☐	☒	The exact sample size (*n*) for each experimental group/condition, given as a discrete number and unit of measurement
☐	☒	A statement on whether measurements were taken from distinct samples or whether the same sample was measured repeatedly
☐	☒	The statistical test(s) used AND whether they are one- or two-sided<br>*Only common tests should be described solely by name; describe more complex techniques in the Methods section.*
☒	☐	A description of all covariates tested
☒	☐	A description of any assumptions or corrections, such as tests of normality and adjustment for multiple comparisons
☐	☒	A full description of the statistical parameters including central tendency (e.g. means) or other basic estimates (e.g. regression coefficient) AND variation (e.g. standard deviation) or associated estimates of uncertainty (e.g. confidence intervals)
☐	☒	For null hypothesis testing, the test statistic (e.g. *F*, *t*, *r*) with confidence intervals, effect sizes, degrees of freedom and *P* value noted<br>*Give P values as exact values whenever suitable.*
☒	☐	For Bayesian analysis, information on the choice of priors and Markov chain Monte Carlo settings
☒	☐	For hierarchical and complex designs, identification of the appropriate level for tests and full reporting of outcomes
☒	☐	Estimates of effect sizes (e.g. Cohen's *d*, Pearson's *r*), indicating how they were calculated

Our web collection on statistics for biologists contains articles on many of the points above.

Software and code

Policy information about availability of computer code

Data collection	Sony MA900 software used to acquire flow cytometry data.
Data analysis	All code to replicate key findings and process data in this study are available at https://github.com/DavidBJaffe/enclone (Git hash 561e3ac). An additional copy of all code for analysis and visualization, including source data, can be found at DOI 10.25452/figshare.plus.20338177. We used version 0.5.175 of enclone, version 1.29.2 of the msa R package, and version 0.1 of the ggseqlogo R package.

For manuscripts utilizing custom algorithms or software that are central to the research but not yet described in published literature, software must be made available to editors and reviewers. We strongly encourage code deposition in a community repository (e.g. GitHub). See the Nature Portfolio guidelines for submitting code & software for further information.

Data

Policy information about availability of data

All manuscripts must include a data availability statement. This statement should provide the following information, where applicable:

- Accession codes, unique identifiers, or web links for publicly available datasets
- A description of any restrictions on data availability
- For clinical datasets or third party data, please ensure that the statement adheres to our policy

All data are publicly available and processed at DOI 10.25452/figshare.plus.20338177. See Methods for informed consent and related information.

Human research participants

Policy information about studies involving human research participants and Sex and Gender in Research.

Reporting on sex and gender	Samples from biological male and female participants were used as part of this work. Additional information is provided in the Extended and Supplementary Information.
Population characteristics	This information is provided in Extended Tables.
Recruitment	Participants were recruited independently by third party vendors, throughout the United States. It is unlikely that particular selection biases were present given the genetic diversity of the individuals in this study, and representation of individuals from multiple continents.
Ethics oversight	An independent IRB providing oversight to the vendor approved the collection protocol and informed consent for all samples for all donors.

Note that full information on the approval of the study protocol must also be provided in the manuscript.

Field-specific reporting

Please select the one below that is the best fit for your research. If you are not sure, read the appropriate sections before making your selection.

☒ Life sciences ☐ Behavioural & social sciences ☐ Ecological, evolutionary & environmental sciences

For a reference copy of the document with all sections, see nature.com/documents/nr-reporting-summary-flat.pdf

Life sciences study design

All studies must disclose on these points even when the disclosure is negative.

Sample size	We chose samples from genetically diverse individuals who were unlikely to be related to each other. As this was an exploratory study and traditional power calculations are not applicable to immune repertoire data, we did not perform or invent a power calculation.
Data exclusions	No data were excluded from the study.
Replication	All central results related to light chain coherence were successfully replicated within and between an additional 25 donors, including an additional 2 donors whose data were independently generated by an group as part of an independent prior study (Phad et al. 2022 Nat. Immunol.). Biological replicates (separate cell aliquots) were used from each of the four main donors.
Randomization	Donors were not randomized to experimental groups as part of this work; this work is not a clinical study and does not describe an intervention-based study. We used samples from 4 separate and genetically unrelated individuals, in additional to using previously published independent data, to assess our results.
Blinding	We did not blind ourselves as part of this study because no randomization was performed.

Reporting for specific materials, systems and methods

We require information from authors about some types of materials, experimental systems and methods used in many studies. Here, indicate whether each material, system or method listed is relevant to your study. If you are not sure if a list item applies to your research, read the appropriate section before selecting a response.

Materials & experimental systems

n/a	Involved in the study	
☐	☒	Antibodies
☒	☐	Eukaryotic cell lines
☒	☐	Palaeontology and archaeology
☒	☐	Animals and other organisms
☒	☐	Clinical data
☒	☐	Dual use research of concern

Methods

n/a	Involved in the study	
☒	☐	ChIP-seq
☐	☒	Flow cytometry
☒	☐	MRI-based neuroimaging

Antibodies

Antibodies used	Described in Methods in subsection "Flow cytometry." Clone, color, isotype, catalog #, host, and manufacturer are provided. All antibodies were used at 1:40 dilution (5 ul per antibody) in a staining volume of 200 ul with a total antibody mass of 1.25 ug.
Validation	All antibodies used in this study are GMP manufactured and have been verified by 3 independent manufacturers and vendors using relative expression (specific staining of known lineage marker-positive cells, no staining of lineage marker-negative cells) with imaging and flow cytometry. All antibodies used in this study have been used extensively in the literature for flow cytometric analysis, including in specialist journals such as Cytometry Part A.

Flow Cytometry

Plots

Confirm that:

☒ The axis labels state the marker and fluorochrome used (e.g. CD4-FITC).

☒ The axis scales are clearly visible. Include numbers along axes only for bottom left plot of group (a 'group' is an analysis of identical markers).

☒ All plots are contour plots with outliers or pseudocolor plots.

☒ A numerical value for number of cells or percentage (with statistics) is provided.

Methodology

Sample preparation	Described in detail in Methods. Briefly, PBMCs from each donor were thawed, washed, stained, analyzed, and sorted using standard protocols and validated antibody panels. See Phad et al. 2022 for methods information related to publicly available data first reported outside of this study.
Instrument	Sony MA-900
Software	Sony MA-900 software; FlowJo used for visualization of data.
Cell population abundance	Reported within figures.
Gating strategy	Reported both in Methods and in Figures.

☒ Tick this box to confirm that a figure exemplifying the gating strategy is provided in the Supplementary Information.
